# First Prospective Cohort Study of Diabetic Retinopathy from Sub-Saharan Africa: High Incidence and Progression of Retinopathy and Relationship to Human Immunodeficiency Virus Infection

**DOI:** 10.1016/j.ophtha.2016.05.042

**Published:** 2016-09

**Authors:** Philip I. Burgess, Simon P. Harding, Marta García-Fiñana, Nicholas A.V. Beare, Gerald Msukwa, Theresa J. Allain

**Affiliations:** 1 Malawi-Liverpool-Wellcome Trust Clinical Research Programme, and Department of Eye and Vision Science, University of Liverpool, Liverpool, England; 2 Department of Eye and Vision Science, University of Liverpool, and St. Paul's Eye Unit, Royal Liverpool University Hospital, Liverpool, England; 3 Department of Biostatistics, University of Liverpool, Liverpool, England; 4 St. Paul's Eye Unit, Royal Liverpool University Hospital, and Department of Eye and Vision Science, University of Liverpool, Liverpool, England; 5 Lions Sight First Eye Unit, Queen Elizabeth Central Hospital, Blantyre, Malawi; 6 College of Medicine, University of Malawi, Blantyre, Malawi

**Keywords:** CI, confidence interval, DR, diabetic retinopathy, ETDRS, Early Treatment of Diabetic Retinopathy Study, HbA1c, glycosylated hemoglobin, HIV, human immunodeficiency virus, LDES, Liverpool Diabetic Eye Study, MDRS, Malawi Diabetic Retinopathy Study, OR, odds ratio, PDR, proliferative diabetic retinopathy, QECH, Queen Elizabeth Central Hospital, STDR, sight-threatening diabetic retinopathy, uACR, urine albumin–creatinine ratio, VA, visual acuity, WHO, World Health Organization, ZCH, Zomba Central Hospital

## Abstract

**Purpose:**

To describe the prevalence, incidence, and progression of retinopathy and to report associations with demographic, clinical, and biochemical variables in people with diabetes in Southern Malawi.

**Design:**

Prospective cohort study.

**Participants:**

Subjects were systematically sampled from 2 primary care diabetes clinics.

**Methods:**

We performed the first prospective cohort study of diabetic retinopathy from Sub-Saharan Africa over 24 months. Visual acuity, glycemic control, blood pressure, human immunodeficiency virus (HIV) status, urine albumin-to-creatinine ratio, hemoglobin, and lipids were assessed. Retinopathy was graded at an accredited reading center using modified Wisconsin grading of 4-field mydriatic photographs.

**Main Outcome Measures:**

Incidence of sight-threatening retinopathy and progression of retinopathy by 2 steps on the Liverpool Diabetic Eye Study Scale.

**Results:**

A total of 357 subjects were recruited to the 24-month cohort study. At baseline, 13.4% of subjects were HIV positive and 15.1% were anemic. The 2-year incidence of sight-threatening diabetic retinopathy (STDR) for subjects with level 10 (no retinopathy), level 20 (background), and level 30 (preproliferative) retinopathy at baseline was 2.7% (95% confidence interval [CI], 0.1–5.3), 27.3% (95% CI, 16.4–38.2), and 25.0% (95% CI, 0–67.4), respectively. In a multivariate logistic analysis, 2-step progression of diabetic retinopathy was associated with glycosylated hemoglobin (odds ratio [OR], 1.27; 95% CI, 1.12–1.45), baseline grade of retinopathy (OR, 1.39; 95% CI, 1.02–1.91), and HIV infection (OR, 0.16; 95% CI, 0.03–0.78). At 2 years, 17 subjects (5.8%) lost ≥15 letters.

**Conclusions:**

Incidence of STDR was approximately 3 times that reported in recent European studies. The negative association of HIV infection with retinopathy progression is a new finding.

The International Diabetes Federation has estimated that the number of adults diagnosed with diabetes in Africa will increase from 12.1 million in 2010 to 23.9 million in 2030.^1^ The prevalence and incidence of sight-threatening diabetic retinopathy (STDR) in developed countries^2^, ^3^, ^4^ and the association with systemic factors, including glycemic control,^5^, ^6^ blood pressure,^7^ and blood lipid levels,^8^ are well documented. No cohort studies have investigated the determinants of severity and progression of diabetic retinopathy (DR) in Sub-Saharan Africa.^9^ In this resource-poor setting, population-specific variables, such as a high burden of infectious disease (including human immunodeficiency virus [HIV] and malaria) and anemia, may affect the spectrum of pathology encountered.

Malawi (population 15.9 million) is one of the poorest countries in the world, with an annual per capita health care expenditure of US$77.^10^ The World Health Organization (WHO) Malawi national STEPwise survey estimated a prevalence of diabetes of 5.6% in adults aged 25 to 64 years, with a similar prevalence in rural and urban areas.^11^ In 2007, our group performed a cross-sectional study using clinical ocular examination to assess grades of retinopathy in patients attending the diabetes clinic at Queen Elizabeth Central Hospital (QECH), Blantyre.^12^, ^13^ We reported a high prevalence of sight-threatening and proliferative retinopathy: 19.6% and 5.7%, respectively. Because of these important findings, we performed the Malawi Diabetic Retinopathy Study (MDRS), a prospective, observational cohort study of patients attending 2 hospital diabetes clinics over 24 months. The study aimed to describe the prevalence, incidence, and progression of DR in Southern Malawi and to investigate the determinants of retinopathy severity and progression in this population. Baseline data from the cohort have been published.^14^

## Methods

### Setting

The QECH in Blantyre is the main teaching hospital in Malawi. It provides primary and secondary care to the urban and semi-urban population of greater Blantyre (∼1.0 million people, 50% adult) and tertiary care to the southern region. Zomba Central Hospital (ZCH) provides primary and secondary care to the Zomba district. The diabetes clinics at QECH and ZCH are the only public sector diabetes clinics in Blantyre and Zomba, with approximately 2000 and 250 registered patients, respectively. The clinics provide free consultation and monitoring (measurement of height and weight, blood pressure, and fasting blood glucose). Medications regularly available free of charge are metformin, glibenclamide, and insulin (lente and soluble), as well as a limited range of antihypertensives.

### Participants

Patient selection has been described.^14^ Briefly, systematic random sampling was used to select subjects from the diabetes clinics at QECH and ZCH between December 2011 and May 2012. Patients attend these clinics for medical management of diabetes; no eye care is provided. The inclusion criterion was a diagnosis of diabetes according to American Diabetes Association criteria.^15^ Exclusion criteria were age <18 years and diagnosis of gestational diabetes according to American Diabetes Association criteria. The diabetes clinics at QECH and ZCH provide predominantly primary diabetes care (primary care for diabetes is nonexistent at the health center level). Central hospitals are tertiary centers that receive referral cases. To effectively exclude referral cases, patients living more than 60 km from the clinic in question and those visiting the clinic for the first time were excluded from the study.

### Procedures

After assessment at baseline, subjects were recalled (by telephone or home visit) at 12 and 24 months. Clinical assessment of subjects in the MDRS has been described.^14^ Briefly, visual acuity (VA) (uncorrected and using pinhole) was measured as the number of letters read on a standard Early Treatment of Diabetic Retinopathy Study (ETDRS) chart. Moderate visual impairment (50–59 letters; equivalent to 6/24 Snellen) and severe visual impairment or blindness (<50 letters; equivalent to 6/36 or worse) were defined according to the WHO.^16^ For each patient with corrected VA <80 letters in the better eye, the primary cause of visual impairment was recorded by the examining clinician (P.I.B.). Subjects were classified as having hypertension according to the WHO definition^11^: taking antihypertensive medication, systolic blood pressure ≥140 mmHg, or diastolic blood pressure ≥90 mmHg. All subjects were offered point-of-care testing for HIV (Malawian national protocol^17^) and hemoglobin level. Thresholds for anemia were set according to WHO guidelines: 130 g/l for men and 120 g/l for women.^18^ Blood samples were assayed for putative biochemical risk factors: fasting glucose, triglycerides, low-density lipoprotein cholesterol, high-density lipoprotein cholesterol, serum creatinine, urine albumin-to-creatinine ratio (uACR), and glycosylated hemoglobin (HbA1c).

Retinopathy and maculopathy were classified by feature-specific grading using definitions established in the Liverpool Diabetic Eye Study (LDES)^19^ (Appendix Fig 1, available at www.aaojournal.org). Dual grading of digital photographic images of four 45° standard fields^19^ was performed by accredited graders at the Liverpool Reading Centre. Sight-threatening diabetic retinopathy was defined as any of the following: moderate preproliferative retinopathy or worse (level 40 to ≥71); macular exudates in a circinate pattern or within 1 disc diameter of the foveal center or clinically significant macular edema (ETDRS definition^20^) (level 3–4: sight-threatening maculopathy); or other diabetes-related retinal vascular disease: central or branch retinal artery occlusion, central or branch retinal vein occlusion. Subjects who met thresholds for scatter or macular laser treatment were treated by 1 ophthalmologist (P.I.B.). Threshold for scatter laser treatment was the ETDRS 4-2-1 rule (4 quadrants of hemorrhages/microaneurysms standard 2A or greater, *or* 2 quadrants of venous beading standard 6A or greater, or 1 quadrant of intraretinal microvascular abnormalities standard 8A or greater). Threshold for macular laser was clinically significant macular edema as defined in the ETDRS^20^ and VA less than 85 ETDRS letters. The majority of deaths in Malawi are not registered. The relatives of deceased subjects were visited at home by a study nurse to confirm the death. Death was recorded if confirmed by a first-degree relative or “traditional authority” (village leader in rural districts).

### Statistical Analysis

Grades of retinopathy were calculated by patient according to the worse or only gradable eye. Visual acuity data were investigated by patient according to the better eye. The primary outcome was progression of DR by 2 or more steps on the LDES severity scale (equates to 1-step progression in both eyes *or* 2-step progression in 1 eye). We constructed a multiple logistic regression model (backward stepwise with probability of removal of 0.2) to determine the odds ratio (OR) and 95% confidence intervals (CIs) for 2-step progression in association with an initial 12 variables: time since diagnosis of diabetes, type of diabetes, baseline grade of DR, mean HbA1c (mean of measurement at baseline and 12 and 24 months), systolic blood pressure, uACR, hemoglobin, high-density lipoprotein cholesterol, triglycerides, HIV status, age, and scatter laser treatment any time between baseline and 24 months. Descriptive analysis showed that uACR did not demonstrate a linear association with probability of a 2-step progression; a logarithmic transformation (base 10) was more suitable. All tests were 2 sided, and a *P* value <0.05 was taken to indicate statistical significance. All calculations were performed using STATA version 12 (StataCorp LP, College Station, TX). The study was approved by the University of Liverpool Research Ethics Committee and the University of Malawi College of Medicine Research Ethics Committee. All participants gave written informed consent.

## Results

**Figure 1:**
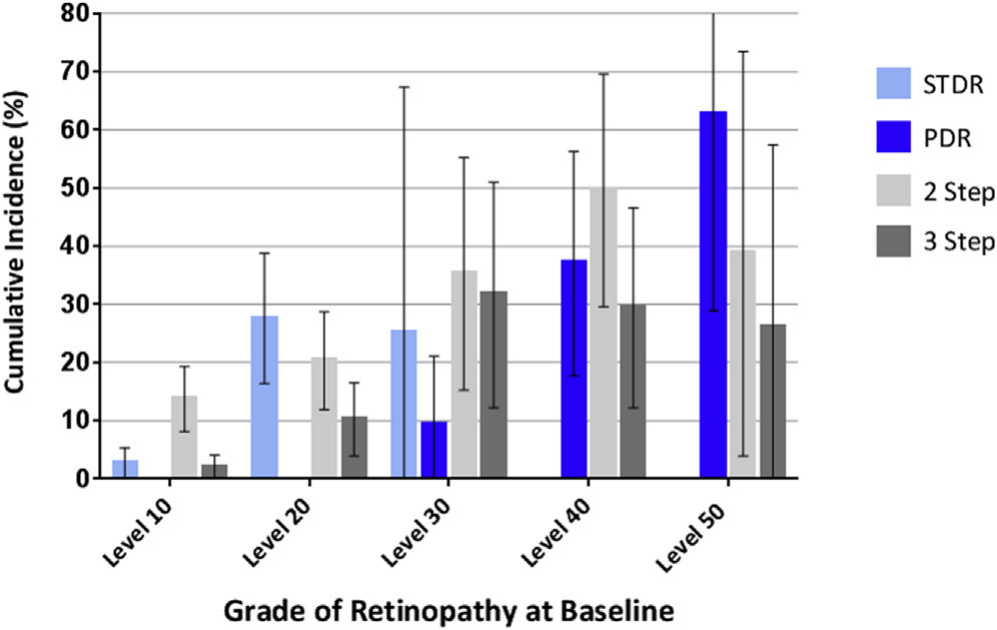
Incidence at 2 years of sight-threatening diabetic retinopathy (STDR) and proliferative diabetic retinopathy (PDR) (level 60+) and of 2 (or more) step and 3 (or more) step progression on the Liverpool Diabetic Eye Study (LDES) scale for subjects in the Malawi Diabetic Retinpathy Study (MDRS) 24-month cohort with level 10 (n = 177), level 20 (n = 94), level 30 (n = 25), level 40 (n = 26), and level 50 (n = 8) retinopathy at baseline. Error bars indicate 95% CI. Classes STDR, PDR, 2-step progression, and 3-step progression are not exclusive, that is, a single subject can develop STDR and PDR and progress by 2 steps on the LDES scale.

Of 357 subjects recruited, 322 were seen for at least 1 further study visit and are included in the progression analysis. A total of 313 subjects (88%) and 295 subjects (83%) were assessed at 12 and 24 months, respectively (Appendix Fig 2, available at www.aaojournal.org). Median time to follow-up was 2.0 years (interquartile range, 1.9–2.1 years). Baseline characteristics of subjects seen at 24 months and those who were not seen are shown in Table 1. A total of 50 subjects (14.0%) were HIV positive (48 at baseline and 2 new diagnoses during the study). Incidence of death in the MDRS cohort at 24 months was 8.0% (95% CI, 5.1–10.9; n = 357; Life Table method). Incidence of death among HIV-positive subjects was 18.1% (95% CI, 7.4–28.8; n = 50). Death during the MDRS was associated with STDR (OR, 2.51; 95% CI, 1.15–5.48; *P* = 0.02; univariate analysis), proliferative DR (PDR; OR, 6.47; 95% CI, 2.51–16.7; *P* = 0.0001), HIV (OR, 3.72; 95% CI, 1.54–9.00; *P* = 0.003), and moderate visual impairment (OR, 8.21; 95% CI, 2.48–27.1; *P* = 0.001). Two (or more) step progression (from baseline) was observed at 12 or 24 months in 69 subjects (OR, 21.4%; 95% CI 16.9–25.9); 3 (or more) step progression was observed in 30 subjects (OR, 9.3%; 95% CI, 6.1–12.5) (this analysis includes both subjects with no retinopathy and those with retinopathy at baseline). Of 225 subjects without STDR at baseline, 23 (OR, 10.2%; 95% CI, 6.3–14.2) developed the condition during the study (Table 2; Appendix Tables 1–3, available at www.aaojournal.org; Fig 1). Of 26 subjects with level 60 DR or more at baseline, at 24 months the following grades of DR were recorded: 7 subjects with level <60, 4 subjects with level 60, and 5 subjects with level >60. Eight subjects died, and 2 subjects were lost to follow-up. Appendix Table 4 (available at www.aaojournal.org) details the number of subjects who were listed for, started, and completed a course of laser treatment during the course of the MDRS.

In univariate analysis, 2-step progression of retinopathy was positively associated with duration of diabetes, baseline grade of DR, scatter laser treatment, HbA1c, and uACR and negatively associated with HIV infection. Higher mean HbA1c and higher baseline grade of retinopathy were risk factors for 2-step progression in multivariate analysis. Human immunodeficiency virus infection was negatively associated with progression of DR (Table 3). This association may have been influenced by the high mortality among subjects with HIV and diabetes. Therefore, we performed a sensitivity analysis. The univariate association of HIV infection with a composite variable of 2-step progression at 24 months or death during the study was not statistically significant (OR, 0.84; 95% CI, 0.41–1.75; *P* = 0.64). Human immunodeficiency virus was not selected by a stepwise procedure for a multiple logistic regression model with the composite term as the dependent variable (data not shown).

A total of 127 subjects (43.0%) lost ≥5 ETDRS letters over the course of the study, of whom 17 (5.8%) lost ≥15 letters. The most common primary causes of visual loss for the 127 subjects who lost ≥5 letters were DR (38.6%), cataract (16.5%), and both DR and cataract (3.9%). Incidence at 24 months of developing moderate visual impairment (50–59 letters; equivalent to 6/24 Snellen) or severe visual impairment or blindness (<50 letters; equivalent to 6/36 or worse) was 0.9% (0–2.0) and 1.5% (0.2–2.8), respectively (Life Table method; n = 322).

## Discussion

We report the first prospective longitudinal study of DR from sub-Saharan Africa. Incidence at 24 months of any DR (new DR) was 38.0%. Incidence of STDR for those with no (level 10), background (level 20), and mild preproliferative (level 30) retinopathy at baseline was 2.7%, 27.3%, and 25.0%, respectively. Higher HbA1c and higher baseline grade of DR were risk factors for progression of retinopathy in multivariate analysis. HIV was negatively associated with progression. Despite the availability of laser treatment, 17 subjects (5.8%) had moderate visual loss (lost ≥15 letters) during the 2 years of the study.

Few high-quality cohort studies are available for comparison from the African or Asian continents. In Mauritius, researchers followed up a population-based study performed in 1992^21^ with a survey of diabetes complications in 1998.^22^ The 6-year incidence of DR and PDR among subjects with diabetes but no DR in the first survey was 23.8% and 0.4%, respectively. The 6-year incidence of PDR among subjects with mild nonproliferative DR (equivalent to level 20 in LDES grading) and moderate nonproliferative DR (equivalent to LDES level 30 or level 40) was 5.2% and 29.4%, respectively. Compared with recent studies of European screening programs, in the MDRS, 2-year progression to STDR from no DR (level 10) and from background DR (level 20) was approximately 3 times (2.7% vs. estimates between 0.5% and 0.8%^2^, ^23^, ^24^, ^25^) and 2.5 times higher (27.3% vs. estimates between 6.4^25^ and 11.2%^2^), respectively.

Differences between our study and recent European work are likely to reflect disparities in diabetic care, ethnicity, access to health services, and presence of comorbidities. The high crude mortality rate (8% over 2 years) among our cohort is comparable to data from Tanzania. In a prospective cohort study, McLarty et al^26^ reported 5-year mortality of 40.5% among those with insulin-dependent diabetes and 19.0% among subjects with non-insulin-dependent diabetes. In contrast, the UK Prospective Diabetes Study^27^ reported all-cause mortality across all study participants at a mean of 10.0 years' follow-up to be 17.9%. In the more recent Action to Control Cardiovascular Risk in Diabetes Study,^28^ follow-up all-cause mortality was 4.5% at a mean of 3.5 years. The association of death with STDR in the MDRS suggests poor glycemic control and the presence of other complications of diabetes. The high mortality indicates the need for improved diabetes care in sub-Saharan Africa, but in the context of our study, it is an important cause of data censoring. As diabetes care improves, the prevalence of retinopathy may (paradoxically) increase because of case survival.

We report a negative association between DR progression and HIV infection, a novel finding. An important potential confounder of this relationship is early diagnosis of diabetes in HIV-positive subjects already attending health facilities. Reduced progression may have been influenced by high mortality in subjects with HIV and diabetes, removing subjects whose DR would have otherwise progressed. This possibility is supported by our sensitivity analysis: Progression of retinopathy was not associated, in univariate or multivariate analysis, with a composite variable of 2-step progression at 24 months or death during the study. Both HIV infection and antiretroviral therapies are associated with a vasculopathy that manifests as increased cardiovascular and cerebrovascular risk.^29^, ^30^ Low-grade proteinuria is highly prevalent in HIV-positive patients taking antiretroviral therapy and is more common in persons with concomitant diabetes.^31^ A real negative association between HIV and DR is biologically plausible, but this finding should be treated with caution.

In the MDRS 24-month cohort study, lower hemoglobin was associated with the presence of STDR at baseline (previously reported^14^) but not with the progression of DR. Cross-sectional (but not cohort) studies have demonstrated an association between the presence of DR and anemia in India^32^, ^33^, ^34^ and China.^35^ Potential confounders of the association between hemoglobin and retinopathy are socioeconomic and nutritional status and decreased erythropoietin production due to diabetic nephropathy. A plausible mechanism for the relationship is impaired oxygen delivery. Whether treatment of anemia reduces diabetic microvascular complications is not known. Iron supplementation has significant potential drawbacks in diabetes: Both high iron level and iron supplementation have been associated with gestational diabetes.^36^, ^37^ To our knowledge, no studies from Africa have reported longitudinal VA data in subjects with diabetes. Although the MDRS was an observational cohort study, subjects could receive medical interventions with the potential for improvement of vision, including laser photocoagulation and cataract surgery. Without these interventions, visual loss is likely to have been greater.

### Study Limitations

We recognize the limitations of our clinic-based study. Barriers to attendance include transportation costs, competing economic tasks (planting and harvesting staple crops), and ignorance of health services. Patients who do not attend clinics may be less likely to be diagnosed with diabetes or to comply with therapy. Conversely, those with established complications may be more likely to attend clinics and participate in research studies. The MDRS included few subjects with diet-controlled diabetes because few of these attend the diabetic clinic.^14^ Although some patients travel long distances to attend clinics, rural subjects are likely to represent a selected subgroup of the rural diabetic population.

The prevalence of diabetes in Africa is increasing rapidly, and there is an urgent need for service provision. Future studies must provide an evidence base for prevention, early detection, and management programs for DR in the region. Our findings represent a baseline against which the efficacy and cost-effectiveness of such interventions can be judged.

## Figures and Tables

**Table 1:** Baseline Demographic, Clinical, and Biochemical Measurements and Retinopathy Grading for 357 Subjects in the Malawi Diabetic Retinopathy 24-Month Cohort Study Categorized by Follow-up: Traced and Assessed at 24 Months (n = 295) or Not Seen at 24 Months (n = 62)

Baseline Characteristic	Subjects Seen at 24 Months **(n = 295)**	Subjects Not Seen at 24 Months **(n = 62)**	*P* Value
Age (yrs), median (IQR)	53.5 (43.4–60.2)	56.2 (48.7–66.0)	0.04∗†
Female sex, n (%)	180 (61.0)	36 (58.1)	0.67‡
Type 1 diabetes, n (%)	29 (9.8)	6 (9.7)	0.99‡
Duration (yrs), median (IQR)	4.1 (1.9–8.1)	4.5 (2.1–8.1)	0.58§
sBP (mmHg), median (IQR)	135 (121–156)	134 (118–159)	0.93†
HbA1c (NGSP %), mean (SD)	7.8 (2.4)	8.0 (3.1)	0.57†
Hb (g/dl), mean (SD)	14.1 (1.8)	13.0 (2.2)	0.0001∗†
HIV positive, n (%)	36 (12.2)	12 (19.4)	0.15‡
LDL cholesterol (mmol/l), mean (SD)	2.49 (0.93)	2.18 (0.99)	0.019∗†
Increased uACR (M >2.5; F >3.5 mg/mmol), n (%)	91 (30.8)	31 (50)	0.005∗‡
Any DR, n (%; 95% CI)	154 (52.2; 46.5–57.9)	25 (40.3; 28.1–52.5)	0.09‡
STDR, n (%; 95% CI)	88 (29.8; 24.6–35.0)	17 (27.0; 16.0–38.1)	0.76‡
Proliferative DR, n (%; 95% CI)	16 (5.4; 2.8–8.0)	10 (16.1; 7.0–25.3)	0.007∗‡

CI = confidence interval; DR = diabetic retinopathy; Hb = hemoglobin; HbA1c = glycosylated hemoglobin; HIV = human immunodeficiency virus; IQR = interquartile range; LDL = low-density lipoprotein; NGSP = National Glycohemoglobin Standardisation Program; sBP = systolic blood pressure; SD = standard deviation; STDR = sight-threatening diabetic retinopathy; uACR = urine albumin-to-creatinine ratio.

∗ Indicates statistical significance.

† Unpaired *t* test.

‡ Fisher exact test.

§ Wilcoxon rank sum test.

**Table 2:** Life Tables Showing Cumulative Yearly Incidence of Development of Grades of Retinopathy and Sight-Threatening Diabetic Retinopathy and of Progression by 2 (or More) and 3 (or More) Steps on the Liverpool Diabetic Eye Study Scale in the Worse Eye of Subjects in the Malawi Diabetic Retinopathy 24-Month Cohort Study and No Retinopathy at Baseline

T	Any Retinopathy				Level 30				Level 40			
N	n	Cumulative Incidence	95% CI	N	n	Cumulative Incidence	95% CI	N	n	Cumulative Incidence	95% CI
1	177	18	10.8%	6.1–15.5	177	6	3.6%	0.8–6.4	177	0	0%	–
2	138	40	38.0%	30.2–45.8	150	3	5.6%	1.9–9.3	156	1	0.7%	0–2.0

T	STDR∗				2+ Step Progression				3+ Step Progression			
N	n	Cumulative Incidence	95% CI	N	n	Cumulative Incidence	95% CI	N	n	Cumulative Incidence	95% CI
1	174	0	0%	–	177	8	4.8%	1.6–8.1	177	2	1.2%	0–2.9
2	154	4	2.7%	0.1–5.3	148	13	13.7%	8.2–19.3	154	1	1.9%	0–4.1

CI = confidence interval; N = number entering time interval; n = new cases diagnosed during year; STDR = sight-threatening diabetic retinopathy; T = time from recruitment (years).

∗ Patients with STDR at baseline were omitted from analysis.

**Table 3:** Risk Factors for Association of Progression of Diabetic Retinopathy by 2 or More Steps on the Liverpool Diabetic Eye Study Scale at 24 Months in the Malawi Diabetic Retinopathy Study (n = 293): Univariate and Multivariate Logistic Regression

	OR	95% CI	*P* Value
Univariate logistic regression			
Duration of diabetes (yrs)	1.05	1.00–1.09	0.042∗
Type 1 diabetes	2.09	0.89–4.89	0.090
Baseline grade of DR	1.48	1.23–1.76	0.001∗
Scatter laser treatment†	3.75	1.90–7.40	0.001∗
Mean HbA1c (NGSP %)	1.28	1.13–1.44	0.001∗
Mean sBP (mmHg)	1.00	0.99–1.02	0.68
log[Mean urine ACR] (mg/mmol)	1.28	1.07–1.52	0.005∗
Mean hemoglobin (g/dl)	0.92	0.76–1.12	0.43
HIV positive	0.20	0.05–0.85	0.029∗
Baseline LDL cholesterol (mmol/l)	0.99	0.72–1.34	0.92
Baseline HDL cholesterol (mmol/l)	2.24	0.98–5.14	0.057
Baseline triglycerides (mmol/l)	0.78	0.57–1.06	0.11
Sex (male)	1.04	0.58–1.87	0.89
Age (yrs)	0.99	0.97–1.01	0.49
Multivariate logistic regression			
Mean HbA1c (NGSP %)	1.27	1.12–1.45	0.001∗
Baseline grade of DR	1.39	1.02–1.91	0.040∗
HIV positive	0.16	0.03–0.78	0.023∗
Type 1 diabetes	2.27	0.87–5.89	0.094
Scatter laser treatment†	1.45	0.43–4.88	0.54

ACR = albumin-to-creatinine ratio; CI = confidence interval; DR = diabetic retinopathy; HbA1c = glycosylated hemoglobin; HDL = high-density lipoprotein; HIV = human immunodeficiency virus; LDL = low-density lipoprotein; NGSP = National Glycohemoglobin Standardisation Program; OR = odds ratio; sBP = systolic blood pressure.

∗ Indicates statistical significance.

† Scatter laser treatment received any time between baseline and 24 months.
