# Supplementary material for: First Prospective Cohort Study of Diabetic Retinopathy from Sub-Saharan Africa: High Incidence and Progression of Retinopathy and Relationship to Human Immunodeficiency Virus Infection
Source: Ophthalmology. 2016 Sep;123(9):1919–25. doi: 10.1016/j.ophtha.2016.05.042 (PMC4994575; doi:10.1016/j.ophtha.2016.05.042)
Supplement: Appendix Table 1 [file mmc1.pdf]

Online Appendix Table 1 Life tables showing cumulative yearly incidence of progression to higher grades of retinopathy and development of sight threatening diabetic retinopathy and of progression by 2 (or more) and 3 (or more) steps on the LDES scale in the worse eye of subjects in the MDRS 24 month cohort study and **level 20** retinopathy at baseline.

	**Level 30**				**Level 40**				**STDR ‡**			
T	N	n	C. Inc.	95% CI	N	n	C. Inc.	95% CI	N	n	C. Inc.	95% CI
1	94	12	13.1	6.2-20.0	94	2	2.2	0-5.2	70	9	13.2	5.2-21.3
2	77	13	27.9	18.6-37.2	87	5	7.9	2.3-13.6	57	9	27.3	16.4-38.2

	**2 Step progression**				**3 Step progression**							
T	N	n	C. Inc.	95% CI	N	n	C. Inc.	95% CI				
1	94	6	6.5	1.5-11.6	94	2	2.2	0-5.2				
2	83	12	20.3	11.9-28.7	87	7	10.2	3.9-16.5				

T = time from recruitment (years); N = number entering time interval; n = new cases diagnosed during year; C. inc. = cumulative incidence (%); CI = confidence interval; STDR = sight threatening diabetic retinopathy. ‡ - patients with STDR at baseline omitted from analysis
