# Supplementary material for: First Prospective Cohort Study of Diabetic Retinopathy from Sub-Saharan Africa: High Incidence and Progression of Retinopathy and Relationship to Human Immunodeficiency Virus Infection
Source: Ophthalmology. 2016 Sep;123(9):1919–25. doi: 10.1016/j.ophtha.2016.05.042 (PMC4994575; doi:10.1016/j.ophtha.2016.05.042)
Supplement: Appendix Table 2 [file mmc2.pdf]

Online Appendix Table 2 Life tables showing cumulative yearly incidence of progression to higher grades of retinopathy and development of sight threatening diabetic retinopathy and of progression by 2 (or more) and 3 (or more) steps on the LDES scale in the worse eye of subjects in the MDRS 24 month cohort study and **level 30** retinopathy at baseline.

	**Level 40**				**Level 50**				**Level 60 +**			
T	N	n	C. Inc.	95% CI	N	n	C. Inc.	95% CI	N	n	C. Inc.	95% CI
1	25	6	25.5	7.9-43.1	25	0	0		25	0	0	
2	16	4	44.2	23.5-65.0	22	1	4.5	0-13.2	22	2	9.1	0-21.1

	**STDR ‡**				**2 Step progression**				**3 Step progression**			
T	N	n	C. Inc.	95% CI	N	n	C. Inc.	95% CI	N	n	C. Inc.	95% CI
1	6	0	0		25	5	21.3	4.8-37.9	25	1	4.3	0-12.5
2	4	1	25.0	0-67.4	17	3	35.2	15.2-55.2	21	6	31.6	12.2-51.0

T = time from recruitment (years); N = number entering time interval; n = new cases diagnosed during year; C. inc. = cumulative incidence (%); CI = confidence interval; STDR = sight threatening diabetic retinopathy. ‡ - those with STDR at baseline omitted from analysis
