# Supplementary material for: First Prospective Cohort Study of Diabetic Retinopathy from Sub-Saharan Africa: High Incidence and Progression of Retinopathy and Relationship to Human Immunodeficiency Virus Infection
Source: Ophthalmology. 2016 Sep;123(9):1919–25. doi: 10.1016/j.ophtha.2016.05.042 (PMC4994575; doi:10.1016/j.ophtha.2016.05.042)
Supplement: Appendix Table 3 [file mmc3.pdf]

Online Appendix Table 3 Life tables showing cumulative yearly incidence of progression to higher grades of retinopathy and of progression by 2 (or more) and 3 (or more) steps on the LDES scale in the worse eye of subjects in the MDRS 24 month cohort study and **level 40** retinopathy at baseline.

	**Level 50**				**Level 60 +**							
T	N	n	C. Inc.	95% CI	N	n	C. Inc.	95% CI				
1	26	1	4.0	0-11.7	26	4	16	2-30				
2	23	2	13.1	0-27.2	20	5	37	18-56				

	**2 Step progression**				**3 Step progression**							
T	N	n	C. Inc.	95% CI	N	n	C. Inc.	95% CI				
1	26	4	16.0	1.6-30.4	26	4	16.0	1.6-30.4				
2	20	8	49.6	29.6-69.6	20	2	24.4	7.2-41.6				

T = time from recruitment (years); N = number entering time interval; n = new cases diagnosed during year; C. inc. = cumulative incidence (%); CI = confidence interval; STDR = sight threatening diabetic retinopathy.
