# Supplementary material for: First Prospective Cohort Study of Diabetic Retinopathy from Sub-Saharan Africa: High Incidence and Progression of Retinopathy and Relationship to Human Immunodeficiency Virus Infection
Source: Ophthalmology. 2016 Sep;123(9):1919–25. doi: 10.1016/j.ophtha.2016.05.042 (PMC4994575; doi:10.1016/j.ophtha.2016.05.042)
Supplement: Appendix Table 4 [file mmc4.pdf]

Online Appendix Table 4 Number of subjects listed for, started and completed a course of laser treatment during the course of the MDRS (December 2011 until May 2014) classified by grade of DR at the start of the study.

**Baseline level of retinopathy**	**n**	**Laser photocoagulation (listed/started/completed course)**		
		**Scatter and macular**	**Scatter alone**	**Macular alone**
**Level 10**	177	0	0	1/0/0
**Level 20**	94	0/0/0	1/1/0	12/11/11
**Level 30**	25	4/4/3	4/4/4	7/7/7
**Level 40**	26	17/16/12	3/3/3	2/2/2
**Level 50**	8	6/6/5	2/2/2	0
**Level 60**	16	13/13/13	3/2/2	0
**Level 70+**	10	8/8/7	2/2/2	0
**90**	1	0	0	0
**Total**	357	48/47/40	15/14/13	22/20/20
