# Supplementary material for: First Prospective Cohort Study of Diabetic Retinopathy from Sub-Saharan Africa: High Incidence and Progression of Retinopathy and Relationship to Human Immunodeficiency Virus Infection
Source: Ophthalmology. 2016 Sep;123(9):1919–25. doi: 10.1016/j.ophtha.2016.05.042 (PMC4994575; doi:10.1016/j.ophtha.2016.05.042)
Supplement: Appendix Figure 1 [file mmc5.pdf]

Online Appendix Figure 1 Levels of retinopathy and maculopathy in the Liverpool Diabetic Eye Study

Level	Definition
**Retinopathy**	
10	No retinopathy
20	Hemorrhages or microaneurysms < ETDRS standard photograph 2A [†]
30	Hemorrhages or microaneurysms ≥ ETDRS standard photograph 2A, and/or 1-6 cotton wool spots
40	Hemorrhages/ microaneurysms ≥ ETDRS standard photograph 2A
	and/or ≥ 6 cotton wool spots
	and/or 1 quadrant venous changes
	and/or IRMA < ETDRS standard photograph 8A
50	IRMA ≥ ETDRS standard photograph 8A
	and/or 2 or more quadrants venous changes
	and/or pre-retinal hemorrhage in absence of proliferation
60	Fibrovascular proliferation
	and/or proliferative retinopathy
70	Diabetic Retinopathy Study high risk characteristics
71	Tractional retinal detachment
72	No fundal view due to vitreous blood
90	Ungradeable due to any other reason e.g. media opacity
**Maculopathy**	
0	No maculopathy
1	Questionable: < 50% certainty of presence of exudate
2	Exudate >1 disc diameter from fixation
3	Circinate ring of exudate within macula >1 disc area in size but not within 1 disc diameter of fixation
4	Exudates within 1 disc diameter of fixation
	and/or presence of clinically significant macular edema

8	Exudates due to other diseases e.g. vein occlusion, choroidal neovascularisation
90	Ungradeable

† Definition of any diabetic retinopathy: ≥ 1 haemorrhage or microaneurysm in either eye. Flame shaped hemorrhages associated with hypertension are discounted. IRMA = intraretinal microvascular abnormalities. ETDRS = Early Treatment Diabetic Retinopathy Study.

Regression of retinopathy: retinopathy graded according to the lesions present. If new vessels have regressed completely after treatment the grader grades the lesions which remain. Peripheral scatter laser scars do not, in themselves, constitute proliferative retinopathy.
