# Supplementary material for: First Prospective Cohort Study of Diabetic Retinopathy from Sub-Saharan Africa: High Incidence and Progression of Retinopathy and Relationship to Human Immunodeficiency Virus Infection
Source: Ophthalmology. 2016 Sep;123(9):1919–25. doi: 10.1016/j.ophtha.2016.05.042 (PMC4994575; doi:10.1016/j.ophtha.2016.05.042)

Online Appendix Figure 2 Flow diagram for subjects in the Malawi Diabetic Retinopathy Study 24 month cohort: enrolment and follow-up at 12 and 24 months.

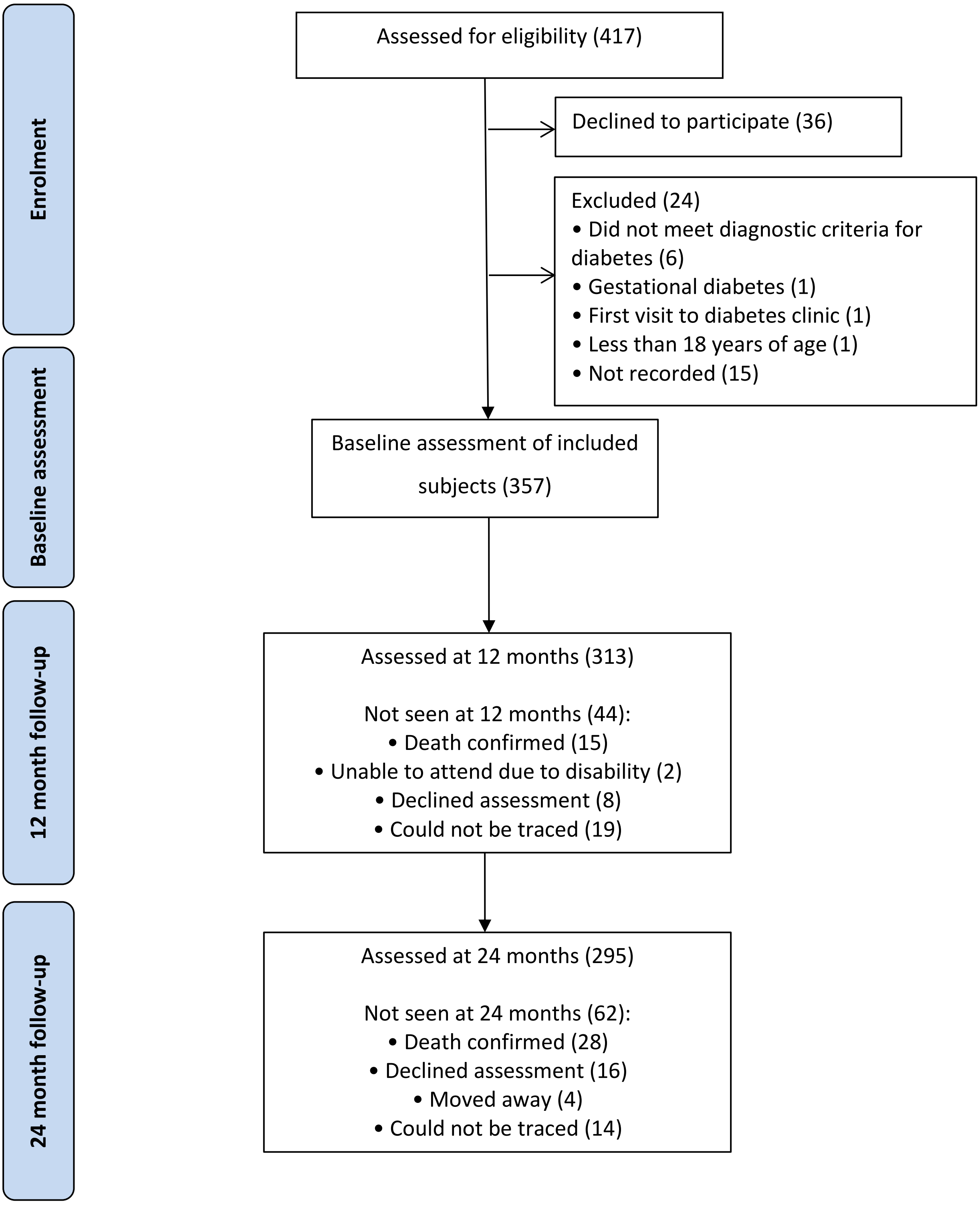

Supplement: Appendix Figure 2 [file mmc6.pdf]
